# Hyperfunctioning distant metastases in high-grade differentiated thyroid carcinoma arising from HRAS-mutated follicular thyroid carcinoma: a case report and literature review

**DOI:** 10.3389/fendo.2026.1817448

**Published:** 2026-04-01

**Authors:** Ji Yong Park, Su Woong Yoo, Sung Sun Kim, A Ram Hong, Jee Hee Yoon, Hee Kyung Kim, Ho-Cheol Kang

**Affiliations:** 1 Department of Internal Medicine, Chonnam National University Medical School, Hwasun, Republic of Korea; 2 Department of Nuclear Medicine, Chonnam National University Medical School, Hwasun, Republic of Korea; 3 Department of Pathology, Chonnam National University Medical School, Hwasun, Republic of Korea

**Keywords:** differentiated thyroid carcinoma, follicular thyroid carcinoma, HRAS mutation, hyperfunctioning metastases, radioactive iodine therapy

## Abstract

**Background:**

Hyperfunctioning distant metastases from differentiated thyroid carcinoma (DTC) are rare but increasingly reported. We report hormone-producing lung and bone metastases from an HRAS-mutated high-grade differentiated thyroid carcinoma (HGDTC) originating from follicular thyroid carcinoma (FTC), with a brief literature review. The metastases showed a marked response to radioactive iodine (RAI).

**Patient findings:**

A 68-year-old woman presented with an enlarging thyroid nodule and multiple pulmonary nodules after starting antithyroid therapy for Graves’ disease. Histopathology confirmed HGDTC arising from FTC, and next-generation sequencing identified an HRAS Gln61Arg mutation. Thyrotoxicosis persisted after total thyroidectomy. A post-therapeutic whole-body radioiodine scan demonstrated iodine-avid pulmonary nodules and a left iliac bone lesion, consistent with hyperfunctioning distant metastases.

**Summary:**

After two RAI treatments, thyroid function shifted from hyperthyroidism to hypothyroidism, and follow-up chest computed tomography showed a significant reduction in pulmonary metastatic lesions.

**Conclusions:**

Hyperfunctioning distant metastases from DTC present diagnostic and therapeutic challenges. This case highlights the consideration of functioning metastases in persistent post-thyroidectomy thyrotoxicosis and demonstrates the potential effectiveness of RAI therapy when metastatic lesions retain iodine avidity. Oncogenic mutations such as HRAS may contribute to the pathophysiology of hormone-producing metastases and provide insights into tumor differentiation and therapeutic responsiveness.

## Introduction

Thyroid nodules with low thyroid-stimulating hormone (TSH) levels are rarely malignant (1), as TSH suppression is thought to limit the initiation and growth of thyroid cancer cells (2). Emerging evidence, however, shows that hyperfunctioning thyroid carcinoma can present as autonomous functioning thyroid nodules or as hyperfunctioning metastatic lesions (2). Fewer than 10% of individuals with differentiated thyroid carcinoma (DTC) develop distant metastases (3), and hyperfunctioning metastases occur in less than 1% (4). Although reports of hyperfunctioning metastases are increasing, cases involving high-grade differentiated thyroid carcinoma (HGDTC) remain exceedingly rare. Here, we report a case of HGDTC with an HRAS mutation in which hyperfunctioning metastases were identified through persistent thyrotoxicosis after total thyroidectomy and showed a marked response to radioiodine therapy.

## Patient presentation

A 68-year-old woman presented with a recently enlarging thyroid nodule and multiple pulmonary nodules found on routine screening. She had been diagnosed with Graves’ disease three months earlier and was taking methimazole. She reported no respiratory or compressive symptoms—such as dyspnea, cough, sputum production, or dysphagia—and no thyrotoxic symptoms, including palpitations, heat intolerance, or weight loss. Chest radiography showed increased leftward tracheal deviation and new multiple lung nodules (**Supplementary Figure 1B**), compared with an image obtained 2 years earlier (**Supplementary Figure 1A**). While taking 7.5 mg of methimazole 7.5 mg daily, her thyroid function tests showed TSH 0.005 μIU/mL (reference range: 0.4–4.8), free T4 (fT4) 3.02 ng/dL (reference range: 0.8–1.71), T3 4.05 ng/mL (reference range: 0.6–1.6), and thyrotropin binding inhibitory immunoglobulin (TBII) 12.9 IU/L (reference range: 0–1.5). Thyroglobulin (Tg) was markedly elevated at 2, 253 ng/mL, with negative anti-thyroglobulin antibody (TgAb) and anti-microsome antibody (TPO Ab). Ultrasonographic (US) examination revealed diffusely hypoechoic thyroid parenchyma with decreased vascularity and a predominantly solid, isoechoic 4.0-cm nodule in the right thyroid lobe (**Supplementary Figure 2**). Fine-needle aspiration (FNA) performed elsewhere showed atypia of undetermined significance (AUS), prompting a core-needle biopsy (CNB), which demonstrated an indeterminate follicular lesion with architectural atypia. Chest computed tomography (CT) confirmed multiple pulmonary nodules, and positron emission tomography-computed tomography (PET-CT) showed fluorodeoxyglucose (FDG)-avid lesions in the right thyroid gland, both lungs, and the left iliac bone (**Supplementary Figures 3A**, **4**). Although the CNB results were inconclusive, total thyroidectomy was performed because of the high suspicion for thyroid malignancy with distant metastases and to manage Graves’ disease. Pathology revealed HGDTC arising from widely invasive follicular thyroid carcinoma (FTC) (**Supplementary Figure 5**), with 5% tumor necrosis and a mitotic count of 5 per 10 high-power fields (HPF). Immunohistochemical staining was positive for thyroid transcription factor-1 (TTF-1), paired box gene 8 (PAX8), cytokeratin, Ki-67 (5%), cyclin D1, Tg, and BCL2. Next-generation sequencing (NGS) identified an HRAS gene mutation (p.Gln61Arg, c.182A>G) with a variant Allele Frequency (VAF) 42.25%.

After total thyroidectomy, she began levothyroxine (LT4) 100 µg daily but soon developed thyrotoxic symptoms. TBII remained elevated at 5.5 IU/L. Symptoms and laboratory evidence of thyrotoxicosis persisted despite dose reduction and eventual discontinuation of LT4. To treat suspected functional metastases, she received radioactive iodine (RAI) therapy at a dose of 150 mCi of I-131. The post-treatment whole-body iodine scan showed significant uptake in pulmonary nodules and the left iliac bone, consistent with metastatic disease (**Supplementary Figure 6A**). Following RAI therapy, Tg was 2, 790 ng/mL with negative TgAb, and TSH was 68.03 μIU/mL. Two weeks later, thyroid function tests indicated hypothyroidism (TSH 1.180 μIU/mL, fT4 0.39 ng/dL, and T3 0.61 ng/mL), and LT4 replacement was restarted at 50 µg and titrated to 125 µg to maintain TSH suppression below 0.1 μIU/mL. Six months later, she received an additional 200 mCi of RAI (I-131). The post-treatment iodine scan again showed significant uptake in iodine-avid lesions in both lungs and the left ilium (**Supplementary Figure 6B**), though less than before. At that time, Tg had decreased to 165 ng/mL; TgAb remained negative; TSH was 55.85 μIU/mL; and TBII had normalized. Over the following 6 months, after the second RAI therapy, serum Tg levels continued to decline, accompanied by a measurable reduction in pulmonary metastases on chest CT (**Supplementary Figures 3 B, C**).

## Discussion

We present a case of HGDTC arising from HRAS-mutant FTC, complicated by hyperfunctioning lung and left iliac bone metastases that caused persistent thyrotoxicosis after total thyroidectomy and responded well to RAI therapy.

Several risk factors have been proposed to contribute to hyperfunctioning primary thyroid carcinoma and functioning metastases. Most reported hyperfunctioning primary thyroid carcinomas are FTCs, and about two-thirds of metastatic hyperfunctioning thyroid carcinomas are also FTCs (2, 4, 5). These patterns suggest that FTC is more likely than papillary thyroid carcinoma (PTC) to give rise to hyperfunctioning tumors, either as primary lesions or as metastases. Tumor size may also play a role, as mean tumor sizes in reported cases often exceed 4 cm (2, 5). In the setting of impaired autoregulation, a large primary or metastatic tumor may produce enough thyroid hormone to cause hyperthyroidism, which is consistent with our case (6). Although most autonomously functioning thyroid nodules are benign, malignancy should still be considered. Because many hyperfunctioning primary or metastatic thyroid carcinomas are follicular in origin, distinguishing follicular adenoma from FTC using FNA or CNB remains difficult, though these procedures can still support evaluation. In individuals who develop thyrotoxicosis after thyroid surgery, metastatic hyperfunctioning thyroid carcinoma should be considered, and whole-body scintigraphy with complementary imaging modalities is recommended to identify metastatic lesions (2).

Associations between gene mutations and the mechanisms driving hyperfunctioning thyroid carcinoma and its metastases have been explored and are summarized in **Table 1**. Activating mutations in the TSH receptor (TSHR) or guanine nucleotide-binding protein alpha s (Gsα) can stimulate cAMP signaling, increasing hormone production and leading to hyperthyroidism (7). Over the past three decades, numerous reports have described hyperfunctioning thyroid carcinoma harboring TSHR mutations, suggesting a pathogenic mechanism of this rare entity (7–17). Niepomniszcze et al. and Shinkai et al. demonstrated that concurrent activating mutations in TSHR and major oncogenic drivers (KRAS G12C or BRAF V600E) may jointly promote tumorigenesis and autonomous hormone production (14, 18). Another proposed mechanism is overexpression of 5-iodothyronine deiodinase (D2), which increases intratumoral conversion of T4 to T3 and may contribute to autonomous thyroid hormone production (4).

**Table 1:** Reported gene mutations in hyperfunctioning thyroid carcinoma (primary or metastatic).

Study (year)	Age/sex	Histology	Tumor size	Gene mutations	HFDM	Treatment	Outcomes
Spambalg (1996) (7)	50/M	FTC	NR	TSHR (Thr632Ile)	Subcutaneous, bones, lungs	Total thyroidectomy + RAI (5600 Mbq, 4 times)	Hyperthyroid→Hypothyroid Died after 2 yrs of follow-up
Russo (1997) (8)	60/F	TIC	5.0 cm	TSHR (Asp633His)	Lungs	Total thyroidectomy + RAI (150, 150, 100, 100 mCi)	Hyperthyroid→Hypothyroid Small uptake in the neck, no uptake in the lung
Russo (1999) (9)	42/F	HCC	7.4 cm	TSHR (Val677Leu)	No	Total thyroidectomy + RAI (50 mCi)	Hyperthyroid→Hypothyroid Disease free
Camacho (2000) (10)	49/F	FTC	3.5 cm	TSHR (Phe486Ile)	No	Near-total thyroidectomy + RAI (150 mCi)	Hyperthyroid→Hypothyroid Disease free
Mircescu (2000) (11)	11/F	PTC	4.0 cm	TSHR (Met453Thr)	No	Total thyroidectomy	No mention of TFT change Disease free
Führer (2003) (12)	59/M	FTC	3.5 cm	TSHR (Asp633Tyr) TSHR (Phe631Ile) (two thyroid nodules)	No	Total thyroidectomy + RAI (154 mCi)→Surgery for NFc. lung metastasis	Subclinical hyperthyroidism →Hypothyroidism No mention of follow-up
Gozu (2004) (13)	NR	PTC	5.0 cm	TSHR (Leu512Arg)	No	Total thyroidectomy + RAI (150 mCi)	Hyperthyroid→Hypothyroid Disease free
Niepomniszcze (2006) (14)	64/F	FTC	6.0 cm	TSHR (Thr620Ile) KRAS (Gly12Cys)	No	Total thyroidectomy + RAI (100 mCi)	Hyperthyroid→Hypothyroid Disease free
Lado-Abeal (2010) (15)	55/F	FTC	4.3 cm	TSHR (Met453Thr) PAX8–PPARγ rearrangement	No	Total thyroidectomy + RAI (100 mCi)	Hyperthyroid→Hypothyroid Disease free
Eszlinger (2014) (16)	17/M	PTC	4.2 cm	TSHR (Thr632Ile)	NR	NR	Subclinical hyperthyroidism No mention of follow-up
	17/F	fvPTC	5.0 cm	PAX8–PPARγ rearrangement	NR	NR	Euthyroid No mention of follow-up
Lu (2016) (22)	79/M	FTC	13.5 cm	NRAS (Gln61Lys) GNAS (Arg201His)	Lung, humerus, T9 spine	Total thyroidectomy + RAI (30, 200 mCi)	Hyperthyroid→Hypothyroid Disease controlled
Blackburn (2018) (17)	12/F	FTC	2.0 cm	TSHR (Ile568Thr)	No	Total thyroidectomy + RAI (dose not reported)	Hyperthyroid→Hypothyroid Disease free
Shinkai (2021) (18)	48/M	PTC	2.6 cm	TSHR (Asp727Glu) BRAF (Val600Glu)	No	Total thyroidectomy + RAI (dose not reported)	Hyperthyroid→Hypothyroid Disease free
Huang (2023) (23)	81/F	FTC	0.7 cm	KRAS (Gln61Arg)	Skull	No Tx (patient's will)	Subclinical hyperthyroidism
Kim (2026)	68/F	HGDTC (from FTC)	4.0 cm	HRAS (Gln61Arg)	Lung, left iliac bone	Total thyroidectomy + RAI (150, 200 mCi)	Hyperthyroid→Hypothyroid PR

F, female; M, male; NR, not reported; TIC, thyroid insular carcinoma; HCC, Hürthle cell carcinoma; FTC, follicluar thyroid carcinoma; PTC, papillary thyroid carcinoma; fvPTC, follicular variant of papillary thyroid carcinoma; HGDTC, high-grade differentiated thyroid carcinoma; TSHR, thyroid-stimulating hormone receptor; PAX8/PPARG, paired box gene 8 and peroxisome proliferator-activated receptor gamma; BRAF, B-Raf proto-oncogene, serine/threonine kinase; NRAS, neuroblastoma RAS viral oncogene homolog; KRAS. Kirsten rat sarcoma viral oncogene homolog; HRAS, Harvey rat sarcoma viral oncogene homolog; GNAS, Guanine Nucleotide-Binding Protein Alpha Subunit; HFDM, hyperfunctioning distant metastases; SC, subcutaneous tissue; RAI, radioactive iodine therapy; NFc, Nonfunctioning; PR, partial response.

Activation of other oncogenes, including RAS, may also contribute to malignant transformation (19–23). RAS mutations activate the PI3K/AKT/mTOR and MAPK signaling pathways, both of which are central to thyroid carcinogenesis (24). In PTC, RAS mutations are typically associated with more indolent, follicular-patterned phenotype, including follicular variant PTC and NIFTP, with lower rates of extrathyroidal extension and lymph node metastasis, and better preservation of thyroid differentiation and radioiodine responsiveness compared with BRAF V600E–mutated tumors (19, 25). In FTC, RAS mutations are the most common genetic alterations, often involving codon 61 of NRAS or HRAS and codons 12 or 13 of KRAS (19). Although RAS mutations have been linked to increased risk of distant metastasis and disease-specific mortality, as reported by Riccio et al. (21), growing evidence suggests that many RAS-mutated DTCs may retain sodium-iodide symporter expression and radioiodine avidity (26). Supporting this, Mu et al. demonstrated that distinct mutational profiles underlie radioiodine uptake patterns, with BRAF and TERT promoter mutations predominating in RAI-refractory tumors. In contrast, most RAS-mutated tumors remain radioiodine-avid despite occasional late-stage loss of radioiodine uptake (9). These findings underscore the molecular heterogeneity of RAI-refractory DTC and suggest that RAS-mutated tumors may maintain relatively preserved responsiveness to radioiodine therapy, reinforcing the importance of mutation-informed treatment strategies.

Consistent with this, HRAS—unlike other RAS isoforms—can be selectively inhibited by farnesyltransferase inhibitors (FTIs). Tipifarnib has demonstrated preclinical antitumor activity in HRAS-driven thyroid malignancies, particularly when combined with MEK inhibitors, and clinical trials evaluating this approach are currently ongoing (27).

In conclusion, this case of HRAS-mutated HGDTC arising from FTC with hyperfunctioning metastases illustrates a rare but clinically important presentation that responded well to RAI therapy. Consistent with prior reports implicating other oncogenic mutations in hormone-producing metastatic disease, this case suggests that HRAS may also contribute to hormone overproduction through shared molecular pathways. Advances in molecular diagnostics may support more individualized treatment strategies that improve disease control while reducing treatment-related morbidity.

## Supplementary material

Supplementary Figure 1 Chest X-ray of the patient. **(A)** Chest X-ray performed two years prior to the initial visit. **(B)** Chest X-ray performed at the initial visit, demonstrating more prominent tracheal deviation to the left (white arrow) and newly detected pulmonary nodules (open arrows).

Supplementary Figure 2 Ultrasonographic findings of the patient. **(A, B)** Diffusely hypoechoic thyroid parenchyma with decreased vascularity was observed. **(C)** A predominantly solid, isoechoic nodule measuring 4.0 cm was identified in the right thyroid lobe.

Supplementary Figure 3 Chest CT images of the patient. **(A)** Preoperative; **(B)** After the first radioactive iodine (RAI) treatment; **(C)** After the second RAI treatment.

Supplementary Figure 4 18F-Fluorodeoxyglucose (18F-FDG) PET-CT images of the patient. **(A)** FDG uptake in the right thyroid gland; **(B)** Multiple areas of FDG uptake in small pulmonary nodules (white arrows); **(C)** FDG uptake in the left ilium.

Supplementary Figure 5 Histological examination (hematoxylin and eosin staining) of the specimen. **(A)** Low-power view showing widely invasive tumor cells (original magnification x100). **(B)** Higher magnification reveals large, pale, eosinophilic tumor cells exhibiting nuclear pleomorphism, irregular nuclear membranes, and nuclear clearing (lower right). Adjacent non-neoplastic thyroid tissue demonstrates hyperplastic follicles with numerous colloid resorption droplets (scalloping) (upper left) (original magnification ×200).

Supplementary Figure 6 Whole-body scintigraphy after radioactive iodine (RAI) therapy. **(A)** After the first RAI therapy; **(B)** After the second RAI therapy.
