# Supplementary figures and images for: Hyperfunctioning distant metastases in high-grade differentiated thyroid carcinoma arising from HRAS-mutated follicular thyroid carcinoma: a case report and literature review
Source: Front Endocrinol (Lausanne). 2026 Apr 1;17:1817448. doi: 10.3389/fendo.2026.1817448 (PMC13079141; doi:10.3389/fendo.2026.1817448)

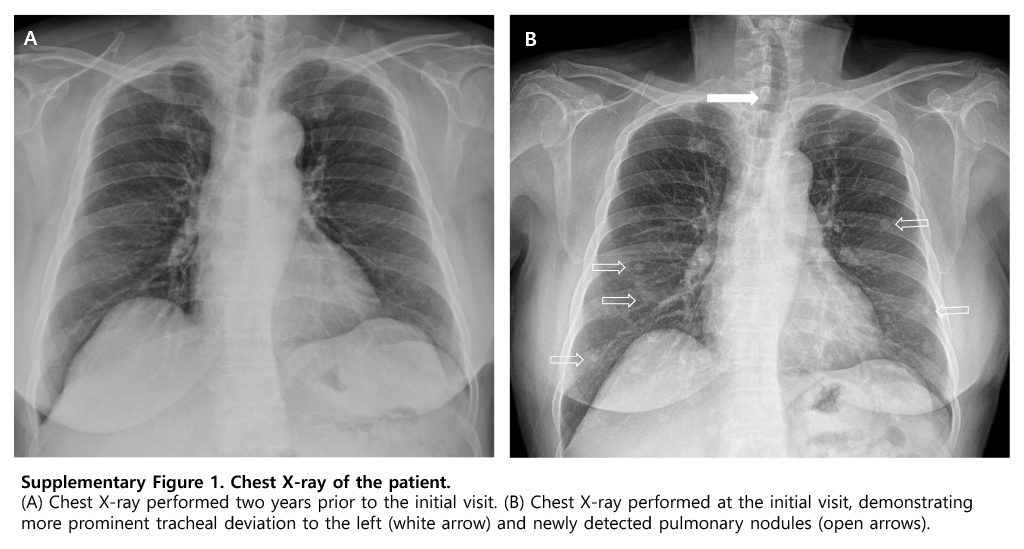

Supplement: Supplementary Figure 1 — Chest X-ray of the patient. (A) Chest X-ray performed two years prior to the initial visit. (B) Chest X-ray performed at the initial visit, demonstrating more prominent tracheal deviation to the left (white arrow) and newly detected pulmonary nodules (open arrows). [file Image1.tiff]

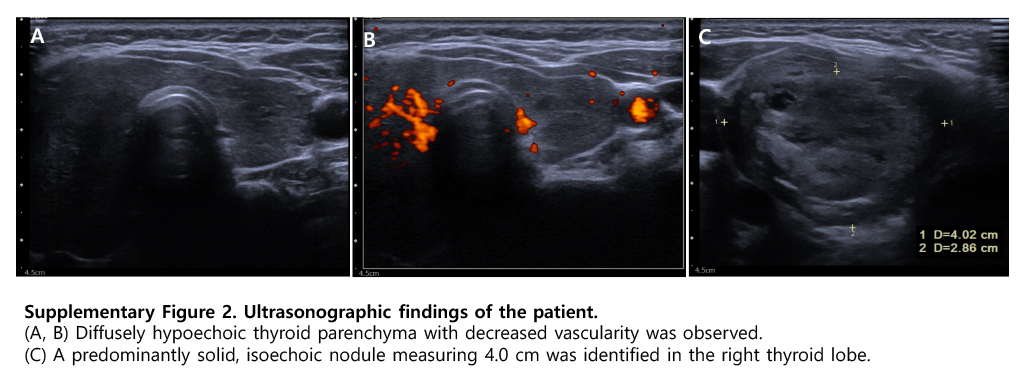

Supplement: Supplementary Figure 2 — Ultrasonographic findings of the patient. (A, B) Diffusely hypoechoic thyroid parenchyma with decreased vascularity was observed. (C) A predominantly solid, isoechoic nodule measuring 4.0 cm was identified in the right thyroid lobe. [file Image2.tiff]

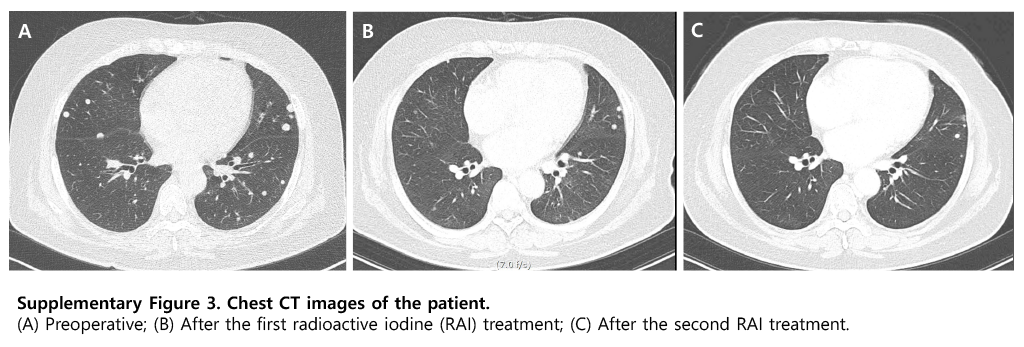

Supplement: Supplementary Figure 3 — Chest CT images of the patient. (A) Preoperative; (B) After the first radioactive iodine (RAI) treatment; (C) After the second RAI treatment. [file Image3.tiff]

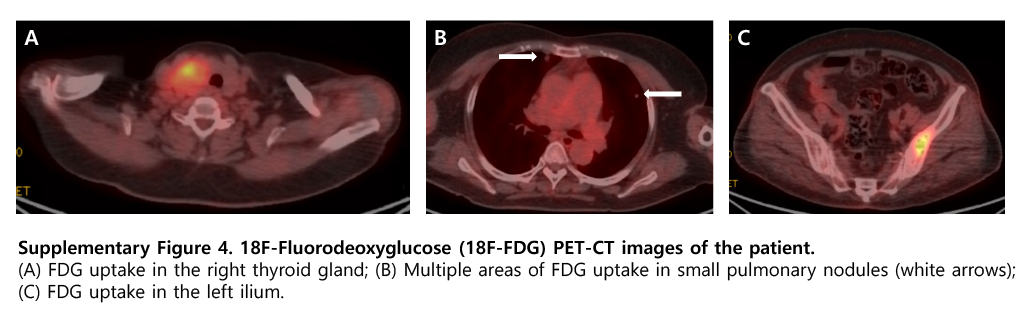

Supplement: Supplementary Figure 4 — 18F-Fluorodeoxyglucose (18F-FDG) PET-CT images of the patient. (A) FDG uptake in the right thyroid gland; (B) Multiple areas of FDG uptake in small pulmonary nodules (white arrows); (C) FDG uptake in the left ilium. [file Image4.tiff]

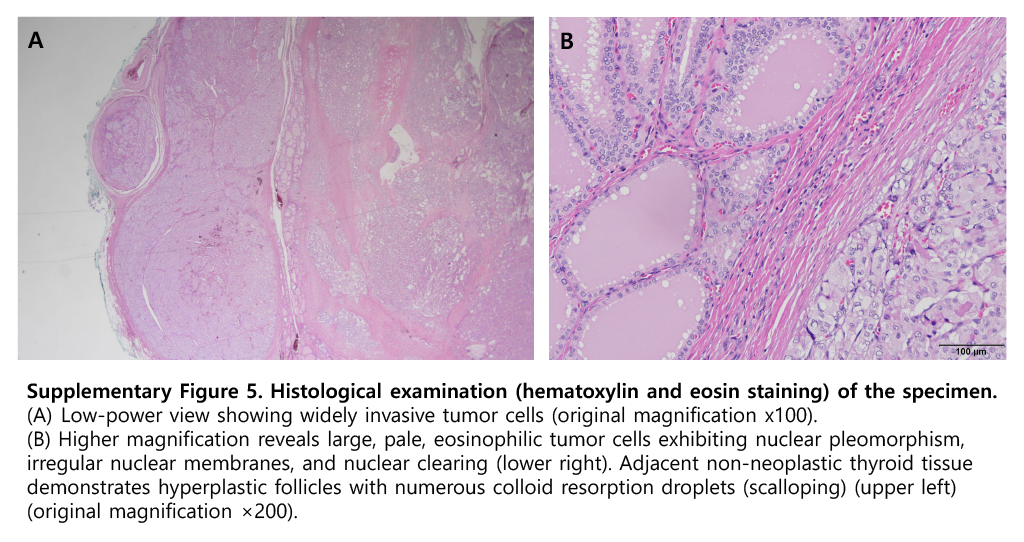

Supplement: Supplementary Figure 5 — Histological examination (hematoxylin and eosin staining) of the specimen. (A) Low-power view showing widely invasive tumor cells (original magnification x100). (B) Higher magnification reveals large, pale, eosinophilic tumor cells exhibiting nuclear pleomorphism, irregular nuclear membranes, and nuclear clearing (lower right). Adjacent non-neoplastic thyroid tissue demonstrates hyperplastic follicles with numerous colloid resorption droplets (scalloping) (upper left) (original magnification ×200). [file Image5.tiff]

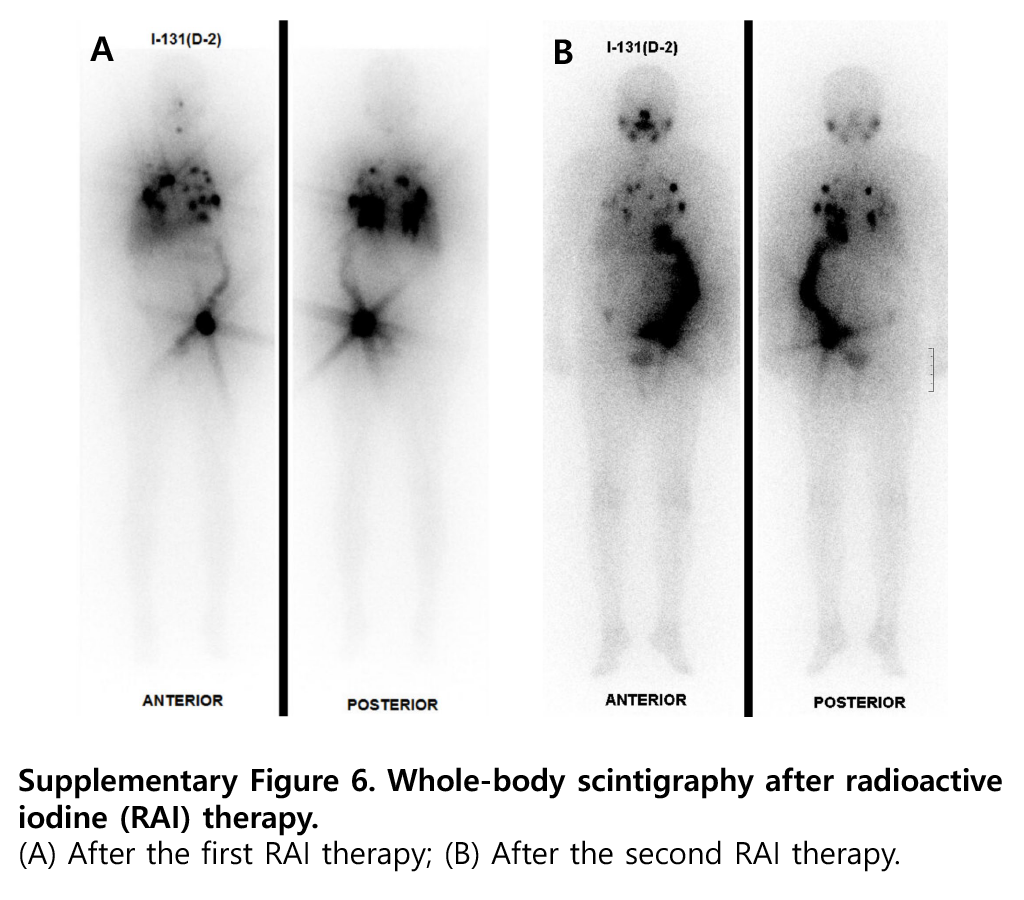

Supplement: Supplementary Figure 6 — Whole-body scintigraphy after radioactive iodine (RAI) therapy. (A) After the first RAI therapy; (B) After the second RAI therapy. [file Image6.tiff]
